# Inverse correlation between Alzheimer’s disease and cancer: implication for a strong impact of regenerative propensity on neurodegeneration?

**DOI:** 10.1186/s12883-014-0211-2

**Published:** 2014-11-14

**Authors:** Jian-Ming Li, Chao Liu, Xia Hu, Yan Cai, Chao Ma, Xue-Gang Luo, Xiao-Xin Yan

**Affiliations:** Department of Anatomy and Neurobiology, Central South University School of Basic Medical Science, Changsha, 410013 Hunan China; Neuroscience Research Center, Changsha Medical University, Changsha, 410219 Hunan China; Department of Neurology, The First Hospital of Changsha, Changsha, 410005 Hunan China; Department of Human Anatomy, Histology & Embryology, Institute of Basic Medical Sciences, Neuroscience Center, Chinese Academy of Medical Sciences; School of Basic Medicine, Peking Union Medical College, Beijing, 100730 China

**Keywords:** Aberrant neuroplasticity, Cell cycle activation, Neurodegeneration, Tumorogenesis

## Abstract

**Background:**

Recent studies have revealed an inverse epidemiological correlation between Alzheimer’s disease (AD) and cancer − patients with AD show a reduced risk of cancer, while cancer survivors are less likely to develop AD. These late discoveries in human subjects call for explorative studies to unlock the underlying biological mechanism, but also may shed new light on conceptual interrogation of the principal pathogenic players in AD etiology.

**Discussion:**

Here we hypothesize that this negative correlation reflects a rebalance of biosynthetic propensity between body systems under the two disease statuses. In normal condition the body cellular systems are maintained homeostatically under a balanced cell degenerative vs. surviving/regenerative propensities, determined by biosynthetic resources for anabolic processing. AD pathogenesis involves neurodegeneration but also aberrant regenerative, or reactive anabolic, burden, while cancer development is driving by uncontrolled proliferation inherent with excessive anabolic activity. The aberrant neural regenerative propensity in AD pathogenesis and the uncontrolled cellular proliferative propensity in cancer pathogeneses can manifest as competitive processes, which could result in the inverse epidemiological correlation seen among the elderly.

**Summary:**

The reduced prevalence of AD in cancer survivors may implicate a strong impact of aberrant neural regenerative burden in neurodegeneration. Further explorative studies into the inverse correlation between AD and cancer should include examinations of the proliferative propensity of tumor cells in AD models, and the development of AD-like neuropathology in cancer models as well as following anti-proliferative drug treatment.

## Background

Alzheimer’s disease (AD) and cancer are major risk factors threatening human health and life especially in the elderly. AD is an age-dependent neurodegenerative disease, while the incidence of cancer is also dramatically increased with age. With the global population aging, AD and cancer become the leading causes of death in most societies across the globe [1,2]. A number of recent studies have revealed a novel inverse correlation in the prevalence of AD vs. cancer in multiple ethnic groups [3–11]. Such a phenomenon points to a certain biological/pathogenic interaction, likely some type of competitive cellular process, between the two disease conditions. Decoding this phenomenon may allow a better understanding of the governing pathogenic factors for AD and/or cancer, and could be also informative for the development of new therapeutical strategies for these diseases. Accordingly, it is of medical relevance to formulate hypothetical model(s) to facilitate mechanistic investigation into the AD/cancer relationship. AD and cancer are both complex conditions with numerous molecular and signaling anomalies occurring in multiple body systems. At the present, little information about the molecular links between the two conditions can be drawn from the human studies. Nonetheless, to understand the inverse correlation it can be benefited from an analysis of the major pathogenic features of the two disease conditions in reference to some basic cell biology principle(s). In this article we first review recent literature documenting the negative association between AD and cancer in human population-based studies. Next we elaborate some major pathological features of AD, with an emphasis on the coexistence of neurodegenerative and aberrant regenerative propensities in the brain. We then discuss the basic aspect of cancer biology, focusing on the influence on body tissue systems by uncontrolled cell proliferation in tumorogenesis. Finally we hypothesize that the reduced AD vs. cancer comorbidity in the elderly may be considered as a result of rebalance of biosynthetic kinetics between body systems. Overall, the inverse epidemiological correlation between AD and cancer may implicate a strong impact of the aberrant neural regenerative activity on the progress of AD-type neurodegeneration.

## Discussion

### Reduced comorbidity between Alzheimer’s disease and cancer in the elderly

The reduced comorbidity of AD vs. cancer in the elderly is recognized fairly lately, for less than ten years. In 2005, Roe et al. reported prospective longitudinal data showing a reduced risk of developing cancer among participants with dementia of the Alzheimer-type (DAT), and a reduced risk of developing DAT among individuals with a history of cancer [3]. A follow-up study by this group confirmed the inverse correlation of cancer with sporadic AD, but not with vascular dementia, among white older adults [4], attracting much attention in the field (see the September issue of Neurology, 2010). In 2012, similar findings are established in a community-based prospective cohort study (1278 participants in total) in the United States [5], and in a case–control study (enrolled 126 AD patients and 252 matched controls) in Italy [6]. In 2013, a population-based longitudinal study (enrolled 1,102 adults with a mean age of 79 years) further shows that individuals with non-melanoma skin cancer have a reduced risk of developing AD [10]. In a large Italy-based cohort study (on more than 1 million Northern Italy residents), the risk of cancer in patients with AD dementia is found to be halved, while the risk of AD dementia in patients with cancer reduced one-third [8]. More recently, a population-based prospective study of 2,627 people without dementia aged 65 years and older shows that individuals with faster cognitive decline have a decreased risk of cancer mortality [11]. Besides western communities, a decreased incidence of overall cancers is observed among Chinese (with 6,960 patients enrolled) with sporadic AD [9]. Thus, the inverse correlation of AD with cancer in the elderly presents among different ethnic groups, and is likely irrelevant to environmental factors.

### Role of aberrant neural regenerative burden in Alzheimer’s disease pathogenesis

The clinical phenotype of AD, largely manifested as cognitive decline, is most likely a result of neurodegeneration. Cerebral atrophy is evident macroscopically in AD, and strong evidence supports that dementia is best correlated to neuronal/synaptic degeneration and dysfunction [12–19]. As discussed in a recent review [20], many genes regulating cell proliferation/survival or apoptosis are altered in AD brains, resulting in a prone-to-death state (AD phenotype). For instances, the tumor suppressor gene p53 may be upregulated in the brains of AD patients [20–22]. The protein interacting with NIMA 1 (Pin1) gene, which is overexpressed in some types of human cancers, is up- or down-regulated in AD brains (depending on brain region) [20,23,24]. The wingless-type murine-mammary tumor virus integration site (Wnt) gene is important for many developmental and adult processes, and a defect in Wnt signaling pathway is suggested to play a role in neurodegeneration in AD [20].

It should be noted that neural regenerative burden is enhanced, and may be also important, in the development of AD. Many “morphoregulatory” molecules that play crucial role during brain development are upregulated in the brains of AD or even prodromal AD subjects [25–29]. These molecules are largely related to anabolic and biosynthetic pathways mediating cell growth/differentiation, neuritic extension, synaptic plasticity, cell adhesion, cytoskeleton and membrane turnover, and signaling control for the above processes [26]. Recent microarray analyses have clearly demonstrated prominent upregulation of numerous genes associated with anabolic/biosynthetic cellular events (transcription, protein biosynthesis, protein trafficking, and turnover), mitochondrial energy generation, as well as synaptic maintenance and function (vesicle trafficking, neurotransmitter receptors, and synaptic structure and stabilization), in the brains of patients with mild cognitive impairment (MCI), a putative preclinical stage of AD [18,19,28,29]. Altered expression of several nerve growth family proteins (e.g., BDNF, TrkB and p75NTR) has been also implicated in degenerative or regenerative pathological events in AD, with some markers apparently elevated in plaque-associated dystrophic neurites [30–32]. Moreover, glial activation is evident in AD brains, which may be also viewed as a part of the rescue attempts in response to neurodegeneration [33–36].

It is perhaps particularly worth noting that the hallmark lesions of AD, amyloid plaques and neurofibrillary tangles, are viewed by some as a part of host responses [27,37]. The β-amyloid precursor protein (APP) plays a trophic role in brain development [38]. Its upregulation in AD brain is particularly evident in dystrophic neurites around plaques [39–42]. β-Secretase-1 (BACE1), the obligatory enzyme initiating APP processing to β-amyloid (Aβ) production, is also apparently elevated in plaque-associated dystrophic axon terminals [43,44]. Further, immunoreactivity of presenilin-1, an active component of the γ-secretase complex liberating Aβ products, is described to accumulate in the dystrophic neurites [45,46]. As with APP, BACE1 and γ-secretase play crucial physiological roles in neuronal and synaptic development and plasticity [47–53]. In line with a regenerative role, the amyloidogenic pathway is activated in response to brain injury and other types of neuronal stress, including in wildtype animal models and humans [36,49,54–57]. In regard to the other principal lesion of AD, neurofibrillary tangles are caused by intraneuronal accumulation of p-tau disassociated from microtubules [37]. Under physiological condition, tau binds to and stabilizes microtubules, while site-specific phosphorylation allows its disassociation from microtubules, which plays a physiological role in cytoskeleton flexibility, neuritic/synaptic plasticity and axonal transportation [58,59]. While the mechanism and consequence of abnormal tau phosphorylation in AD remains unclear [60], p-tau proteins are richly expressed in the brain during development [61].

While the molecular and cellular changes categorized above as anabolic events in AD pathogenesis may be activated to facilitate cell proliferation, survival and regeneration to compensate neuronal death, these events should not be generally regarded beneficial or neuronal protective, at least not over the entire disease course. As some of the cell surviving/regenerative responses (e.g., inflammation, Aβ production and tau phosphorylation) eventually yield toxic/detrimental effects on neuronal systems, neurodegenerative changes are exacerbated [25–27,54]. Similarly, the ectopic activation of cell cycle-mediated events in mature neurons may end-up with neuronal damage and death [62,63]. As a matter of fact, the notion that maladapted regenerative responses become self-propelling forces driving vicious pathological cycles has been long proposed for AD as well as other neurological diseases [25–27].

The AD/cancer inverse correlation would imply that there exists a certain common biological process underlying a “mutual-competition” for the development of either AD or cancer. Based on this assumption, it appears that the maladapted neural regenerative propensity in AD pathogenesis better parallels or “matches” with the uncontrolled proliferative propensity in cancer development (see next section), given that cellular replication and regeneration/repair use the same anabolic/biosynthetic machinery.

### Metabolic impact of tumorogenesis on body systems

Cancer development involves complex biological deregulations in cellular and tissue systems [64]. At the present, no clear information can yet be drawn from the aforementioned epidemiological studies regarding the molecular underpinning of the AD/cancer inverse correlation. However, the most remarkable biological feature of cancer pathogenesis involves uncontrolled tumorous growth. We therefore elaborate a potential impact of this change on body systems including the brain. The anabolic activity driving cancer development must be fueled by energy and nutrition supply, which is available with limitation in the body. Thus, cancer development would be associated with a “hijack” of the body’s bioenergetic and nutritional resources, and a deprivation thereof, from normal tissue systems. Such an overall biological effect can be explicated by the cachexia phenomenon in cancer patients [65]. Cachexia is suggestive of a shift of anabolic resources to cancer tissue at the expense of catabolic activity in other body systems. While cachexia is most evident among end-stage cancer patients, it is reasonable to consider that biological modulations with a deprivation of anabolic supply by the tumorous tissue from other cellular compartments must occur before the wasting syndrome is overtly manifested.

As major medical attention to cancer patients is life-saving, the extent of a potential rebalance in anabolic kinetics between tissue compartments, especially the effect thereof on brain metabolism and function, has scarcely been evaluated, therefore remains unclear to date. However, the brain receives 15-20% of the body’s blood supply, while it weighs about 2% of the total body mass. It is expectable that a dominated anabolic cellular processing inherent with tumorogenesis would very likely place a considerable or substantial deprivation effect on the biosynthetic activity in the brain given its great demand for and dependency on metabolic resource. Accordingly, such an effect would likely influence the aberrant regenerative propensity described above in the course of AD pathogenesis.

### Potential interplay between Alzheimer’s disease and cancer pathogenesis

**Figure 1:**
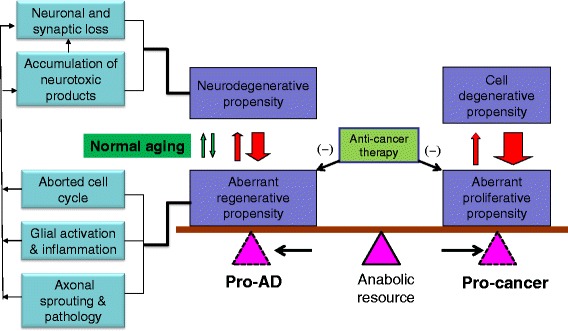
**Schematic illustration of a hypothetic biological model for the inverse epidemiological association between Alzheimer’s disease (AD) and cancer in the elderly.** In AD, degenerative as well as aberrant proliferative/regenerative responses are enhanced in the brain relative to normal aging. The former manifests as neuronal death and synaptic loss, which may be caused by neurotoxic molecules (e.g., Aβ and phosphorylated tau, inflammatory factors) overproduced in the brain. The later could be derived from maladapted regenerative changes including cell cycle reentry, glial proliferation and aberrant neuroplasticity, which can lead to the production of the above-mentioned neurotoxic products or may directly cause neuronal death. The vicious neuropathological cycle is maintained in the brain at the expense of body’s biosynthetic resources (energy and nutrients) for other cellular system, which can reduce the propensity for tumorogenesis because of a mutual competition (the balance point shifting to a pro-AD pathogenic trend). An opposite flow of the body’s biosynthetic resources occurs in the event of cancer development. The propensity of uncontrolled cancer cell replication deprives the body’s biosynthetic resources, including that for the anabolic activity driving the aberrant regenerative burden (the balance point shifting to pro-cancer pathogenic tendency). Consistent with the above biological mechanism, anti-cancer drugs may mitigate AD-type neuropathology, given that their anti-proliferative pharmacological efficacy would relieve the burden of aberrant neural regeneration, and thus slows down vicious pathogenic cycle.

Having analyzed the factors relevant to AD pathogenesis and cancer development, we propose the potential cellular and metabolic alterations that could underlie the inverse epidemiological association between AD and cancer seen among the elderly (Figure 1). In the development of AD, cell/neuronal death and surviving machineries are activated relative to normal aging. Therefore, the biological burden or propensity for neural degenerative and regenerative processing is enhanced in the brain. The degenerative events include neuronal death and synaptic loss, which may be induced by the accumulated endogenous neurotoxic substances such as Aβ and p-tau. The regenerative propensities include aberrant cell cycle activation, glial proliferation, inflammation and aberrant neuronal sprouting, which can generate neurotoxic products (Aβ, inflammatory cytokines, etc.) that worsen neurodegeneration. As a result, a vicious pathogenic cycle of neural degeneration and aberrant regeneration forms and speeds up in the brain with disease progression. This cycle is maintained at the expense of body’s biosynthetic resources. Because of the anatomic arrangement for a prioritized blood supply to the brain (as noted in the above paragraph), the biosynthetic resources tend to be shifted to primarily support the AD pathogenesis, and consequently, deprived from other cellular systems including for supporting tumorogenesis. In the case of cancer development, an opposite shift of the body’s biosynthetic resources occurs. Thus, the uncontrolled anabolic activity with tumorogenesis, driving by malignant genetic modulations, is capable of forming an abnormal locus that overrides the body’s normal adjusting system for the brain-prioritized distribution of biosynthetic resources. This can cause a deprivation effect on anabolism in the brain, especially the biosynthetic activities maintaining the aberrant regenerative propensity, which may result in a delay or slowdown of the development of AD-type pathology (Figure 1).

The biosynthetic resource referred above as the balance point includes the nutritional and bioenergetic systems supporting cellular processes in general. The nutritional components imply to substrates and catalytic elements (e.g., vitamins and metals participating enzymatic activity) for molecular synthesis for such as nucleotides, proteins and lipids. Regarding bioenergetic metabolism, several review articles have discussed this topic in relevance to the inverse epidemiological correlation of cancer with AD and other neurodegenerative diseases [20,66–68], therefore we only briefly note here. Specifically, for cancer development it has been well established that tumorogenesis involves a shift from oxidative phosphorylation towards aerobic glycolysis, a phenomenon known as the Warburg effect [69]. For AD development, it is commonly believed that the pathologically accumulated cellular products (Aβ, p-tau, proinflammatory molecules) cause neuronal and synaptic degeneration (i.e., according to the amyloid, tau and chronic inflammation hypotheses) [27,54]. Alternatively, AD may be considered as a metabolic disease that is primarily caused by bioenergetic failure from the progressive effects of aging-related damage (entropy, oxidative damage, somatic mutations), with a compensatory upregulation of energy metabolism by affected neurons, namely an inverse Warburg effect [66,67]. It should be noted that human imaging studies have consistently revealed an overall reduced oxidative bioenergetic metabolism in the brain during normal aging, and in prodromal and definitive AD patients, especially in cerebral regions critically involved in cognitive function [70]. Further studies would be needed to characterize the extent of inverse Warburg effect occurring in the brains of AD subjects or during AD-like neuropathological process, including its onset, progression and cellular substrates, and also in AD animal models that show typical AD type pathology (assuming that there is a link between the metabolic effect and neuropathology). Importantly, emerging evidence suggests that the Warburg-type energy metabolism epitomizes a physiological signature of cell proliferation and differentiation [69,71,72]. Based on this advance in understanding of general cell biology, one might expect that the aberrant regenerative cellular processes described above for AD pathogenesis, including the maladapted neuronal and axonal regenerative attempts and glial activation [69], could be associated with a Warburg-type energy metabolism.

In addition to the above proposed biological modification, the use of anti-tumor drugs perhaps should be also put into consideration, as a potential confounding factor, especially in the interpretation of the reduced comorbidity of AD among cancer survivors (since these patients would have or may be continuously received anti-proliferative treatments). In this regard, it is important to note that a recent large population-based cohort study has shown that the risk of developing AD, vascular dementia or other dementias is significantly lowered in patients receiving chemotherapy compared to those without chemotherapy, although chemotherapy can cause drug-induced dementia [7]. Further, in an animal study it is reported that chronic low-dose administration of carmustine strikingly reduces amyloid plaque burden in a mouse model of AD [73]. Moreover, some anti-cancer drugs, e.g., paclitaxel and epothilone D, which stabilize microtubules and inhibit mitosis, can attenuate tauopathy and axonal pathology [74]. It is reasonable to speculate that these anti-cancer drugs would elicit an inhibitory effect on cell proliferative activities in the brain of the experimental animals. The beneficial effect by cancer therapeutics seen in the AD animal models appears in line with the notion that anti-proliferative modulation can attenuate AD-type neuropathology perhaps by relieving the regenerative burden. In sum, these human and animal pharmacological data, although still fairly preliminary in nature at this stage, appear to be particularly coherent with the biological hypothesis we put forward in this work (Figure 1).

## Summary

AD has become a global healthcare crisis due to population aging, while no promising preventive and therapeutic strategies are currently available for this disease. The inverse association between AD and cancer discovered in human population-based studies deserves a great attention in disease biology and translational research. Additional epidemiological studies should be carried out to further confirm this relationship, with stratified analyses applied to determine whether this phenomenon links to different cancer types and the use of cancer drugs. Experimental studies should start to explore how AD conditions may influence tumorogenesis, and the cellular and molecular mechanism thereof. Vice versa, additional studies should be conducted to determine whether, and if so, how, condition of cancer or anti-cancer drugs modulate AD-type neuropathology. The concept raised in this work may be informative for additional clinical and explorative studies to unlock the biological basis underlying the novel inverse correlation between AD and cancer seen in humans. Our hypothesis has put together multiple pieces of information (e.g., the major neuropathological events and some genetic/molecular modulations in AD, the Warburg-type energy metabolism in physiological and pathological cell replication and regeneration, and the potential effect of anti-proliferative chemotherapy) that appear to be coherent in regard to the mutual-competitive nature of AD vs. cancer development. Obviously, much additional effort is needed to develop other conceptual models in order to fully unlock the mystery behind the AD/cancer inverse association.
